# {2-[(Benzylphenyl­phosphanyl-κ*P*)methyl]phenyl-κ*C* ^1^}iodidobis(trimethyl­phos­phane)cobalt(II)

**DOI:** 10.1107/S1600536811022288

**Published:** 2011-06-25

**Authors:** Jiong Jia, Chenggen Wang, Nazhen Liu, Xiaoyan Li

**Affiliations:** a School of Chemistry and Chemical Engineering, Shandong University, Jinan 250100, People’s Republic of China

## Abstract

In the title compound, [Co(C_20_H_18_P)I(C_3_H_9_P)_2_], the Co^II^ atom has a distorted square-pyramidal geometry, the base of which is comprised of two *trans* PMe_3_ groups, an I atom, and a C atom of the benzyl group. This benzyl group is tethered to the P atom at the apex of the pyramid, thereby forming a five-membered chelated Co—C—C—C—P ring.

## Related literature

The structures of related cobalt(II) compounds have been reported by Klein *et al.* (2003 ▶). For other related compounds, see: Xu *et al.* (2009 ▶). For synthesis details, see: Klein & Karsch (1975 ▶).
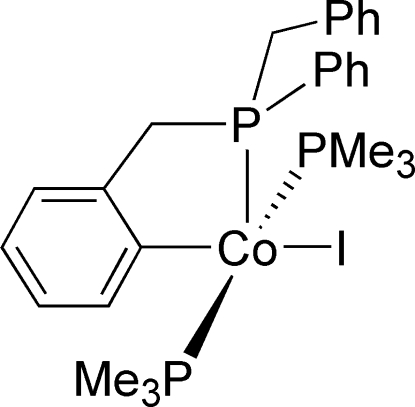


## Experimental

### 

#### Crystal data

[Co(C_20_H_18_P)I(C_3_H_9_P)_2_]
*M* *_r_* = 627.29
Monoclinic, 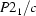

*a* = 16.9282 (19) Å
*b* = 10.6239 (12) Å
*c* = 16.7590 (18) Å
β = 109.120 (2)°
*V* = 2847.7 (5) Å^3^
*Z* = 4
Mo *K*α radiation
μ = 1.87 mm^−1^
*T* = 273 K
0.25 × 0.23 × 0.20 mm

#### Data collection

Bruker APEXII CCD diffractometer
Absorption correction: multi-scan (*SADABS*; Bruker, 2001 ▶) *T*_min_ = 0.653, *T*_max_ = 0.707
13751 measured reflections
5010 independent reflections
3195 reflections with *I* > 2σ(*I*)
*R*_int_ = 0.048

#### Refinement

*R*[*F* ^2^ > 2σ(*F* ^2^)] = 0.046
*wR*(*F* ^2^) = 0.126
*S* = 1.02
5010 reflections
280 parameters
H-atom parameters constrained
Δρ_max_ = 0.77 e Å^−3^
Δρ_min_ = −0.84 e Å^−3^

### 

Data collection: *APEX2* (Bruker, 2004 ▶); cell refinement: *SAINT-Plus* (Bruker, 2001 ▶); data reduction: *SAINT-Plus*; program(s) used to solve structure: *SHELXS97* (Sheldrick, 2008 ▶); program(s) used to refine structure: *SHELXL97* (Sheldrick, 2008 ▶); molecular graphics: *SHELXTL* (Sheldrick, 2008 ▶); software used to prepare material for publication: *SHELXTL*.

## Supplementary Material

Crystal structure: contains datablock(s) global, I. DOI: 10.1107/S1600536811022288/pk2321sup1.cif

Structure factors: contains datablock(s) I. DOI: 10.1107/S1600536811022288/pk2321Isup2.hkl

Additional supplementary materials:  crystallographic information; 3D view; checkCIF report

## Figures and Tables

**Fig. 1.**
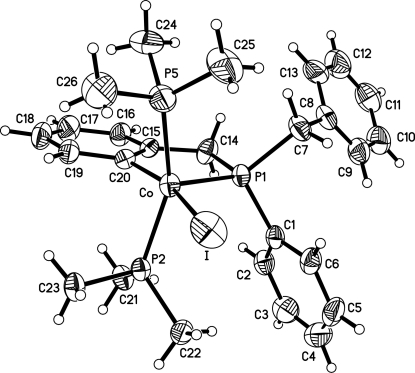
The molecular structure of the title compound with the displacement ellipsoids shown at the 30% probability level.

## supplementary crystallographic information

### Comment

Reaction of low valent complexes of Co(PMe_3_)_4_ with dibenzylphenylphosphine and 4,4'-diiodobiphenyl afforded the title compound. The coordination of P1 and C20 forms a five membered chelated ring.

In the title molecule (Fig. 1) The Co atom lies at the center of the base of a square-based pyramid in which P5 atom and P2 atom are located in *trans* positions. The P1 atom, which occupies the apex of the square-based pyramid is shifted significantly towards C20. The square-pyramidal coordination of Co includes three P-donor atoms, one I atom and one C atom. A five membered chelated ring is formed by C20, C15, C14, P1 and Co. The Co—I distance is 2.6133 (9) Å.

### Experimental

Standard vacuum techniques were used in manipulations of volatile and air sensitive material. Tetrakis(trimethylphosphine)cobalt(0) was prepared according to the literature procedure reported by Klein & Karsch (1975). Other chemicals were used as purchased.

A solution of Co(PMe_3_)_4_ (0.46 g, 1.22 mmol) in 20 ml of THF was added to dibenzylphenylphosphine (0.35 g, 1.21 mmol) in 20 ml of THF.  After stirring at room temperature for 48 h, 4,4'-diiodobiphenyl (0.40 g, 0.99 mmol) was added. The solution turned reddish-brown. THF was evaporated *in vacuo* and the residue was extrated using pentane. Crystallization at 4°C afforded brown crystals suitable for X-ray diffraction analysis (yield 0.12 g, 39%), m.p.: 127°C.

### Refinement

All H atoms on C were placed in calculated positions with a C—H bond distances of 0.93, 0.96 or 0.97 Å and *U*_iso_(H) = 1.2*U*_eq_ or 1.5*U*_eq_(C_Me_) of the carrier atom.

### Crystal data

**Table:**

[Co(C_20_H_18_P)I(C_3_H_9_P)_2_]	*F*(000) = 1268
*M**_r_* = 627.29	*D*_x_ = 1.463 Mg m^−^^3^
Monoclinic, *P*2_1_/*c*	Mo *K*α radiation, λ = 0.71073 Å
Hall symbol: -P 2ybc	Cell parameters from 2338 reflections
*a* = 16.9282 (19) Å	θ = 2.4–23.4°
*b* = 10.6239 (12) Å	µ = 1.87 mm^−^^1^
*c* = 16.7590 (18) Å	*T* = 273 K
β = 109.120 (2)°	Block, brown
*V* = 2847.7 (5) Å^3^	0.25 × 0.23 × 0.20 mm
*Z* = 4	

### Data collection

**Table:**

Bruker APEXII CCD diffractometer	5010 independent reflections
Radiation source: fine-focus sealed tube	3195 reflections with *I* > 2σ(*I*)
graphite	*R*_int_ = 0.048
φ and ω scans	θ_max_ = 25.0°, θ_min_ = 2.3°
Absorption correction: multi-scan (*SADABS*; Bruker, 2001)	*h* = −20→20
*T*_min_ = 0.653, *T*_max_ = 0.707	*k* = −12→6
13751 measured reflections	*l* = −19→19

### Refinement

**Table:**

Refinement on *F*^2^	Primary atom site location: structure-invariant direct methods
Least-squares matrix: full	Secondary atom site location: difference Fourier map
*R*[*F*^2^ > 2σ(*F*^2^)] = 0.046	Hydrogen site location: inferred from neighbouring sites
*wR*(*F*^2^) = 0.126	H-atom parameters constrained
*S* = 1.02	*w* = 1/[σ^2^(*F*_o_^2^) + (0.0573*P*)^2^ + 0.6966*P*] where *P* = (*F*_o_^2^ + 2*F*_c_^2^)/3
5010 reflections	(Δ/σ)_max_ < 0.001
280 parameters	Δρ_max_ = 0.77 e Å^−^^3^
0 restraints	Δρ_min_ = −0.84 e Å^−^^3^

### Special details

**Table:**

Geometry. All e.s.d.'s (except the e.s.d. in the dihedral angle between two l.s. planes) are estimated using the full covariance matrix. The cell e.s.d.'s are taken into account individually in the estimation of e.s.d.'s in distances, angles and torsion angles; correlations between e.s.d.'s in cell parameters are only used when they are defined by crystal symmetry. An approximate (isotropic) treatment of cell e.s.d.'s is used for estimating e.s.d.'s involving l.s. planes.
Refinement. Refinement of *F*^2^ against ALL reflections. The weighted *R*-factor *wR* and goodness of fit *S* are based on *F*^2^, conventional *R*-factors *R* are based on *F*, with *F* set to zero for negative *F*^2^. The threshold expression of *F*^2^ > σ(*F*^2^) is used only for calculating *R*-factors(gt) *etc*. and is not relevant to the choice of reflections for refinement. *R*-factors based on *F*^2^ are statistically about twice as large as those based on *F*, and *R*-factors based on ALL data will be even larger.

### Fractional atomic coordinates and isotropic or equivalent isotropic displacement parameters (Å^2^)

**Table:**

	*x*	*y*	*z*	*U*_iso_*/*U*_eq_	
I	0.33889 (3)	0.67309 (5)	0.26272 (2)	0.0932 (2)	
Co	0.29167 (4)	0.61526 (6)	0.10240 (4)	0.0459 (2)	
P2	0.42352 (8)	0.64403 (12)	0.10397 (8)	0.0440 (3)	
P1	0.22980 (8)	0.78563 (12)	0.02902 (8)	0.0441 (3)	
P5	0.18479 (11)	0.50693 (16)	0.11529 (13)	0.0783 (5)	
C21	0.4455 (3)	0.6527 (5)	0.0050 (3)	0.0550 (14)	
H21A	0.4148	0.5882	−0.0325	0.082*	
H21B	0.5043	0.6407	0.0157	0.082*	
H21C	0.4291	0.7337	−0.0204	0.082*	
C13	0.0175 (4)	0.8953 (7)	−0.1112 (5)	0.086 (2)	
H13	−0.0013	0.8137	−0.1085	0.104*	
C15	0.2366 (3)	0.5969 (4)	−0.0816 (3)	0.0484 (12)	
C1	0.2959 (3)	0.9251 (4)	0.0415 (3)	0.0477 (12)	
C6	0.3132 (3)	0.9901 (5)	0.1171 (3)	0.0594 (14)	
H6	0.2865	0.9680	0.1555	0.071*	
C14	0.2054 (4)	0.7297 (5)	−0.0801 (3)	0.0592 (14)	
H14A	0.1454	0.7322	−0.1083	0.071*	
H14B	0.2314	0.7848	−0.1105	0.071*	
C16	0.2281 (3)	0.5412 (5)	−0.1587 (3)	0.0603 (14)	
H16	0.2055	0.5873	−0.2081	0.072*	
C19	0.2973 (3)	0.4065 (5)	−0.0132 (4)	0.0614 (15)	
H19	0.3210	0.3595	0.0356	0.074*	
C20	0.2743 (3)	0.5314 (5)	−0.0051 (3)	0.0483 (12)	
C22	0.4874 (3)	0.7737 (5)	0.1617 (3)	0.0615 (14)	
H22A	0.4816	0.7801	0.2167	0.092*	
H22B	0.4694	0.8508	0.1314	0.092*	
H22C	0.5450	0.7585	0.1676	0.092*	
C8	0.0858 (4)	0.9407 (6)	−0.0452 (4)	0.0621 (15)	
C4	0.4117 (5)	1.1194 (6)	0.0790 (6)	0.094 (2)	
H4	0.4519	1.1826	0.0920	0.113*	
C17	0.2525 (4)	0.4190 (6)	−0.1632 (4)	0.0776 (18)	
H17	0.2463	0.3827	−0.2154	0.093*	
C18	0.2863 (4)	0.3501 (6)	−0.0903 (4)	0.0781 (19)	
H18	0.3015	0.2665	−0.0930	0.094*	
C2	0.3359 (3)	0.9608 (5)	−0.0148 (3)	0.0577 (14)	
H2	0.3240	0.9193	−0.0663	0.069*	
C3	0.3929 (4)	1.0575 (6)	0.0048 (5)	0.0819 (19)	
H3	0.4191	1.0807	−0.0339	0.098*	
C23	0.4849 (4)	0.5073 (5)	0.1543 (3)	0.0603 (14)	
H23A	0.4566	0.4319	0.1288	0.090*	
H23B	0.4915	0.5074	0.2135	0.090*	
H23C	0.5389	0.5108	0.1473	0.090*	
C10	0.0705 (5)	1.1317 (6)	−0.1237 (5)	0.094 (2)	
H10	0.0894	1.2128	−0.1280	0.113*	
C5	0.3710 (4)	1.0888 (6)	0.1354 (5)	0.083 (2)	
H5	0.3820	1.1337	0.1855	0.099*	
C7	0.1291 (3)	0.8590 (5)	0.0285 (4)	0.0660 (16)	
H7A	0.0911	0.7921	0.0312	0.079*	
H7B	0.1401	0.9089	0.0794	0.079*	
C11	0.0043 (5)	1.0865 (9)	−0.1863 (5)	0.095 (2)	
H11	−0.0226	1.1357	−0.2332	0.113*	
C25	0.1172 (5)	0.5788 (8)	0.1691 (5)	0.112 (3)	
H25A	0.0958	0.6573	0.1424	0.168*	
H25B	0.1491	0.5935	0.2273	0.168*	
H25C	0.0716	0.5232	0.1658	0.168*	
C9	0.1111 (4)	1.0601 (6)	−0.0531 (4)	0.0763 (18)	
H9	0.1563	1.0941	−0.0106	0.092*	
C24	0.1075 (4)	0.4487 (8)	0.0183 (6)	0.123 (3)	
H24A	0.0847	0.5181	−0.0188	0.185*	
H24B	0.0634	0.4064	0.0318	0.185*	
H24C	0.1338	0.3909	−0.0092	0.185*	
C12	−0.0222 (5)	0.9686 (9)	−0.1794 (5)	0.104 (3)	
H12	−0.0682	0.9367	−0.2218	0.125*	
C26	0.2174 (6)	0.3598 (7)	0.1733 (7)	0.143 (4)	
H26A	0.2537	0.3145	0.1498	0.214*	
H26B	0.1691	0.3096	0.1690	0.214*	
H26C	0.2467	0.3782	0.2316	0.214*	

### Atomic displacement parameters (Å^2^)

**Table:**

	*U*^11^	*U*^22^	*U*^33^	*U*^12^	*U*^13^	*U*^23^
I	0.1045 (4)	0.1402 (5)	0.0456 (3)	0.0115 (3)	0.0391 (2)	0.0004 (2)
Co	0.0559 (4)	0.0433 (4)	0.0431 (4)	0.0047 (3)	0.0222 (3)	0.0068 (3)
P2	0.0519 (8)	0.0442 (8)	0.0370 (7)	0.0066 (6)	0.0161 (6)	0.0003 (5)
P1	0.0512 (8)	0.0419 (7)	0.0414 (7)	0.0064 (6)	0.0183 (6)	0.0016 (6)
P5	0.0731 (11)	0.0646 (11)	0.1111 (14)	0.0027 (9)	0.0492 (11)	0.0230 (10)
C21	0.062 (3)	0.060 (3)	0.052 (3)	0.011 (3)	0.031 (3)	0.009 (3)
C13	0.048 (4)	0.083 (5)	0.118 (6)	0.015 (4)	0.013 (4)	0.011 (4)
C15	0.045 (3)	0.043 (3)	0.054 (3)	−0.003 (2)	0.013 (2)	−0.012 (2)
C1	0.055 (3)	0.039 (3)	0.047 (3)	0.013 (2)	0.014 (3)	0.010 (2)
C6	0.069 (4)	0.053 (3)	0.049 (3)	0.009 (3)	0.011 (3)	−0.004 (3)
C14	0.080 (4)	0.048 (3)	0.043 (3)	0.001 (3)	0.011 (3)	−0.002 (2)
C16	0.068 (4)	0.058 (4)	0.049 (3)	0.001 (3)	0.011 (3)	−0.012 (3)
C19	0.062 (4)	0.047 (3)	0.068 (4)	0.005 (3)	0.012 (3)	−0.003 (3)
C20	0.044 (3)	0.044 (3)	0.056 (3)	−0.002 (2)	0.015 (3)	−0.002 (2)
C22	0.066 (4)	0.049 (3)	0.062 (3)	0.002 (3)	0.012 (3)	−0.003 (3)
C8	0.052 (4)	0.067 (4)	0.073 (4)	0.019 (3)	0.029 (3)	0.005 (3)
C4	0.075 (5)	0.051 (4)	0.131 (7)	−0.001 (3)	−0.002 (5)	0.025 (5)
C17	0.082 (4)	0.077 (5)	0.066 (4)	0.004 (4)	0.012 (3)	−0.033 (4)
C18	0.085 (5)	0.054 (4)	0.088 (5)	0.008 (3)	0.017 (4)	−0.020 (4)
C2	0.068 (4)	0.046 (3)	0.061 (3)	0.011 (3)	0.023 (3)	0.013 (3)
C3	0.079 (5)	0.065 (4)	0.105 (6)	0.002 (4)	0.034 (4)	0.031 (4)
C23	0.071 (4)	0.050 (3)	0.058 (3)	0.016 (3)	0.019 (3)	0.005 (3)
C10	0.086 (5)	0.064 (5)	0.128 (7)	0.024 (4)	0.029 (5)	0.021 (4)
C5	0.095 (5)	0.046 (4)	0.081 (5)	0.013 (4)	−0.006 (4)	−0.010 (3)
C7	0.062 (4)	0.069 (4)	0.078 (4)	0.012 (3)	0.038 (3)	0.006 (3)
C11	0.082 (5)	0.105 (7)	0.090 (6)	0.037 (5)	0.019 (5)	0.022 (5)
C25	0.111 (6)	0.130 (7)	0.131 (7)	0.014 (5)	0.088 (6)	0.032 (5)
C9	0.071 (4)	0.062 (4)	0.087 (5)	0.021 (3)	0.014 (4)	0.004 (4)
C24	0.071 (5)	0.120 (7)	0.189 (9)	−0.025 (5)	0.055 (6)	−0.028 (6)
C12	0.076 (5)	0.109 (7)	0.106 (6)	0.029 (5)	0.000 (4)	−0.007 (5)
C26	0.126 (7)	0.087 (6)	0.237 (12)	0.011 (5)	0.088 (8)	0.084 (7)

### Geometric parameters (Å, °)

**Table:**

I—Co	2.6132 (8)	C22—H22B	0.9600
Co—C20	1.943 (5)	C22—H22C	0.9600
Co—P5	2.2137 (18)	C8—C9	1.359 (8)
Co—P2	2.2445 (15)	C8—C7	1.491 (8)
Co—P1	2.2454 (14)	C4—C3	1.350 (10)
P2—C21	1.817 (5)	C4—C5	1.378 (10)
P2—C22	1.822 (5)	C4—H4	0.9300
P2—C23	1.824 (5)	C17—C18	1.376 (8)
P1—C1	1.826 (5)	C17—H17	0.9300
P1—C14	1.837 (5)	C18—H18	0.9300
P1—C7	1.872 (5)	C2—C3	1.373 (8)
P5—C24	1.829 (8)	C2—H2	0.9300
P5—C26	1.828 (7)	C3—H3	0.9300
P5—C25	1.839 (7)	C23—H23A	0.9600
C21—H21A	0.9600	C23—H23B	0.9600
C21—H21B	0.9600	C23—H23C	0.9600
C21—H21C	0.9600	C10—C11	1.348 (10)
C13—C12	1.364 (10)	C10—C9	1.385 (9)
C13—C8	1.397 (8)	C10—H10	0.9300
C13—H13	0.9300	C5—H5	0.9300
C15—C16	1.384 (7)	C7—H7A	0.9700
C15—C20	1.415 (7)	C7—H7B	0.9700
C15—C14	1.510 (7)	C11—C12	1.348 (10)
C1—C2	1.384 (7)	C11—H11	0.9300
C1—C6	1.387 (7)	C25—H25A	0.9600
C6—C5	1.399 (8)	C25—H25B	0.9600
C6—H6	0.9300	C25—H25C	0.9600
C14—H14A	0.9700	C9—H9	0.9300
C14—H14B	0.9700	C24—H24A	0.9600
C16—C17	1.373 (7)	C24—H24B	0.9600
C16—H16	0.9300	C24—H24C	0.9600
C19—C18	1.381 (8)	C12—H12	0.9300
C19—C20	1.401 (7)	C26—H26A	0.9600
C19—H19	0.9300	C26—H26B	0.9600
C22—H22A	0.9600	C26—H26C	0.9600
			
C20—Co—P5	88.38 (15)	H22A—C22—H22C	109.5
C20—Co—P2	85.48 (14)	H22B—C22—H22C	109.5
P5—Co—P2	155.65 (6)	C9—C8—C13	116.8 (6)
C20—Co—P1	87.73 (15)	C9—C8—C7	122.5 (6)
P5—Co—P1	102.89 (6)	C13—C8—C7	120.6 (6)
P2—Co—P1	100.39 (5)	C3—C4—C5	119.9 (7)
C20—Co—I	164.38 (15)	C3—C4—H4	120.1
P5—Co—I	90.37 (6)	C5—C4—H4	120.1
P2—Co—I	89.30 (4)	C16—C17—C18	119.9 (5)
P1—Co—I	107.72 (4)	C16—C17—H17	120.0
C21—P2—C22	100.6 (3)	C18—C17—H17	120.0
C21—P2—C23	101.9 (2)	C17—C18—C19	119.3 (5)
C22—P2—C23	101.9 (3)	C17—C18—H18	120.3
C21—P2—Co	119.77 (19)	C19—C18—H18	120.3
C22—P2—Co	121.62 (19)	C3—C2—C1	120.3 (6)
C23—P2—Co	108.09 (19)	C3—C2—H2	119.8
C1—P1—C14	107.8 (2)	C1—C2—H2	119.8
C1—P1—C7	100.7 (2)	C4—C3—C2	121.3 (7)
C14—P1—C7	102.9 (3)	C4—C3—H3	119.4
C1—P1—Co	115.64 (15)	C2—C3—H3	119.4
C14—P1—Co	101.37 (17)	P2—C23—H23A	109.5
C7—P1—Co	126.81 (18)	P2—C23—H23B	109.5
C24—P5—C26	100.8 (4)	H23A—C23—H23B	109.5
C24—P5—C25	101.4 (4)	P2—C23—H23C	109.5
C26—P5—C25	102.8 (4)	H23A—C23—H23C	109.5
C24—P5—Co	117.3 (3)	H23B—C23—H23C	109.5
C26—P5—Co	112.5 (3)	C11—C10—C9	121.5 (7)
C25—P5—Co	119.4 (3)	C11—C10—H10	119.3
P2—C21—H21A	109.5	C9—C10—H10	119.3
P2—C21—H21B	109.5	C4—C5—C6	119.8 (6)
H21A—C21—H21B	109.5	C4—C5—H5	120.1
P2—C21—H21C	109.5	C6—C5—H5	120.1
H21A—C21—H21C	109.5	C8—C7—P1	116.5 (4)
H21B—C21—H21C	109.5	C8—C7—H7A	108.2
C12—C13—C8	121.2 (7)	P1—C7—H7A	108.2
C12—C13—H13	119.4	C8—C7—H7B	108.2
C8—C13—H13	119.4	P1—C7—H7B	108.2
C16—C15—C20	120.9 (5)	H7A—C7—H7B	107.3
C16—C15—C14	119.0 (5)	C10—C11—C12	118.7 (7)
C20—C15—C14	120.1 (4)	C10—C11—H11	120.7
C2—C1—C6	118.8 (5)	C12—C11—H11	120.7
C2—C1—P1	124.2 (4)	P5—C25—H25A	109.5
C6—C1—P1	116.6 (4)	P5—C25—H25B	109.5
C1—C6—C5	119.8 (6)	H25A—C25—H25B	109.5
C1—C6—H6	120.1	P5—C25—H25C	109.5
C5—C6—H6	120.1	H25A—C25—H25C	109.5
C15—C14—P1	110.7 (3)	H25B—C25—H25C	109.5
C15—C14—H14A	109.5	C8—C9—C10	120.8 (7)
P1—C14—H14A	109.5	C8—C9—H9	119.6
C15—C14—H14B	109.5	C10—C9—H9	119.6
P1—C14—H14B	109.5	P5—C24—H24A	109.5
H14A—C14—H14B	108.1	P5—C24—H24B	109.5
C17—C16—C15	121.0 (5)	H24A—C24—H24B	109.5
C17—C16—H16	119.5	P5—C24—H24C	109.5
C15—C16—H16	119.5	H24A—C24—H24C	109.5
C18—C19—C20	123.0 (6)	H24B—C24—H24C	109.5
C18—C19—H19	118.5	C11—C12—C13	121.1 (8)
C20—C19—H19	118.5	C11—C12—H12	119.5
C19—C20—C15	115.8 (5)	C13—C12—H12	119.5
C19—C20—Co	124.2 (4)	P5—C26—H26A	109.5
C15—C20—Co	120.1 (4)	P5—C26—H26B	109.5
P2—C22—H22A	109.5	H26A—C26—H26B	109.5
P2—C22—H22B	109.5	P5—C26—H26C	109.5
H22A—C22—H22B	109.5	H26A—C26—H26C	109.5
P2—C22—H22C	109.5	H26B—C26—H26C	109.5
